# Expediting citation screening using PICo-based title-only screening for identifying studies in scoping searches and rapid reviews

**DOI:** 10.1186/s13643-017-0629-x

**Published:** 2017-11-25

**Authors:** John Rathbone, Loai Albarqouni, Mina Bakhit, Elaine Beller, Oyungerel Byambasuren, Tammy Hoffmann, Anna Mae Scott, Paul Glasziou

**Affiliations:** Centre for Research in Evidence Based Practice, Bond University, Gold Coast, Australia

**Keywords:** Systematic review, Rapid review, Scoping search, Expediting citation screening, Title screening, Semi-automation, PICo

## Abstract

**Background:**

Citation screening for scoping searches and rapid review is time-consuming and inefficient, often requiring days or sometimes months to complete. We examined the reliability of PICo-based title-only screening using keyword searches based on the PICo elements—Participants, Interventions, and Comparators, but not the Outcomes.

**Methods:**

A convenience sample of 10 datasets, derived from the literature searches of completed systematic reviews, was used to test PICo-based title-only screening. Search terms for screening were generated from the inclusion criteria of each review, specifically the PICo elements—Participants, Interventions and Comparators. Synonyms for the PICo terms were sought, including alternatives for clinical conditions, trade names of generic drugs and abbreviations for clinical conditions, interventions and comparators. The MeSH database, Wikipedia, Google searches and online thesauri were used to assist generating terms. Title-only screening was performed by five reviewers independently in Endnote X7 reference management software using OR Boolean operator. Outcome measures were recall of included studies and the reduction in screening effort. Recall is the proportion of included studies retrieved using PICo title-only screening out of the total number of included studies in the original reviews. The percentage reduction in screening effort is the proportion of records not needing screening because the method eliminates them from the screen set.

**Results:**

Across the 10 reviews, the reduction in screening effort ranged from 11 to 78% with a median reduction of 53%. In nine systematic reviews, the recall of included studies was 100%. In one review (oxygen therapy), four of five reviewers missed the same included study (median recall 67%). A post hoc analysis was performed on the dataset with the lowest reduction in screening effort (11%), and it was rescreened using only the intervention and comparator keywords and omitting keywords for participants. The reduction in screening effort increased to 57%, and the recall of included studies was maintained (100%).

**Conclusions:**

In this sample of datasets, PICo-based title-only screening was able to expedite citation screening for scoping searches and rapid reviews by reducing the number of citations needed to screen but requires a thorough workup of the potential synonyms and alternative terms. Further research which evaluates the feasibility of this technique with heterogeneous datasets in different fields would be useful to inform the generalisability of this technique.

**Electronic supplementary material:**

The online version of this article (10.1186/s13643-017-0629-x) contains supplementary material, which is available to authorized users.

## Background

There is no universal definition of what constitutes a scoping search although various criteria have been proposed [1–3]. In general, scoping searches are useful to attain a preliminary assessment of the size and scope of research literature and to help assess the feasibility of conducting research, including determining whether clinical questions have previously been evaluated, or are up-to-date, and for estimating time frames and budgetary considerations. Similarly, rapid reviews have no universally agreed-upon definition but typically are a form of knowledge synthesis where some components of the systematic review process are simplified or omitted to produce information in a timely manner [4].

Scoping searches and rapid reviews both seek knowledge using a less formalised and rigorous methodology compared with systematic reviews. Rapid reviews attempt to expedite the process by modifying tasks that traditional systematic reviews eschew due to the concerns over data loss [5]. Some tasks that are modified include literature searching, which may be expedited by restricting the number of databases searched [4], restricting by date range and language types [5] or omitting grey literature searches. Other strategies include restricting the number of personnel who screen studies, abstract data and assess risk of bias [4].

Citation screening of the title and abstract is time-consuming because of the large number of citations typically retrieved (the average retrieval from a PubMed search produces 17,284 citations [6]) and is imprecise with often over 98% of citations from systematic searches excluded after title/abstract and full text screening [7–16]. Titles of published studies typically incorporate the main components of a study design which can be categorised into the PICo components (Participants, Intervention, and Comparator, but not the Outcome). Therefore, screening restricted to the title field using the PICo components and the associated synonyms should identify the corpus of relevant studies whilst also being more precise, due to the constrained screening method. The aim of this study was to evaluate the feasibility of PICo-based title-only screening for scoping searches and rapid reviews by measuring the reduction in screening effort and the maintenance of recall of relevant records.

## Methods

A convenience sample of 10 datasets derived from the literature searches of completed systematic reviews were used to test the PICo-based title-only screening. Seven datasets [7, 9–12, 14, 16] available to the authors were used, and an additional three datasets [8, 13, 15] were created by replicating the search strategy from the published reviews. These three reviews were selected prima facie based on being intervention studies that contained adequately reported search strategies and study inclusion details. Duplicate records were removed from the datasets to provide a more homogeneous corpus so that any benefits from reduced screening effort would not be artificially inflated by the presence of duplicate records. We used a convenience sample of five reviewers, (three clinicians and two non-clinicians) based at the Centre for Research in Evidence-Based Practice, Bond University, to assess the reliability and reproducibility of PICo-based title-only screening. All reviewers were familiar with evidence-based practice and systematic review methodology. Four reviewers had no prior knowledge of the datasets used and were unaware of the titles of included studies. One reviewer was a co-author on three of the review datasets; however, the PICo screening terms were derived from the inclusion criteria of the reviews and were not based upon potential recollection of titles of included studies.

Each reviewer independently compiled a list of search terms derived from the inclusion criteria of the reviews, specifically the (P) Participants, (I) Interventions and (C) Comparators but not the Outcomes. PICo synonyms including drug trade names and alternate names for clinical conditions were sought in the MeSH database, Wikipedia, online thesauri and Google searches. Typically, three to four synonyms were generated for each term (see Appendix 1), but there was no restriction on the number of terms used. For further examples, see Additional file 1. Keywords with both British and American spellings were used, and keywords with different suffixes were truncated using an asterisk. PICo-based title-only screening was performed in Endnote X7 reference management software using ‘OR’ Boolean operator (see Appendix 2).

Outcome measures (Table 1) were the recall of included studies and the reduction in screening effort (RSE). Recall is the proportion of included studies retrieved using PICo title-only screening out of the total number of included studies in the original reviews. The percentage reduction in screening effort is the proportion of records that do not need to be screened i.e. those records which are eliminated from the set needing screening by the PICo screening method. This was reported individually for each reviewer and as the median value across the five scores. A post hoc analysis was performed with one of the datasets (Parkinson’s) to examine the impact of screening using only keywords for the (I) Intervention and (C) Comparator and omitting keywords for (P) Participants.

**Table 1:** Outcome measures - Recall of included studies and the reduction in screening effort (RSE)

Recall of included studies	
$$ \mathrm{recall}\ \left(\%\right)=\frac{\mathrm{number}\ \mathrm{of}\ \mathrm{included}\ \mathrm{studies}\ \mathrm{retrieved}}{\mathrm{total}\ \mathrm{number}\ \mathrm{of}\ \mathrm{included}\ \mathrm{studies}}\times 100 $$	
Reduction in screening effort (RSE)	
$$ \mathrm{Precision}=\frac{\mathrm{Number}\ \mathrm{of}\ \mathrm{included}\ \mathrm{studies}\ \mathrm{retrieved}}{\mathrm{Total}\ \mathrm{number}\ \mathrm{of}\ \mathrm{records}\ \mathrm{retrieved}} $$	
$$ \mathrm{Screening}\ \mathrm{effort}\ \left(\mathrm{SE}\right)=\frac{1}{\mathrm{Precision}} $$	
$$ \mathrm{Reduction}\ \mathrm{in}\ \mathrm{screening}\ \mathrm{effort}\ \mathrm{RSE}\ \left(\%\right)=\frac{{\mathrm{SE}}_{\mathrm{method}{1}^{\dagger }}-{\mathrm{SE}}_{\mathrm{method}{2}^{\ddagger }}}{{\mathrm{SE}}_{\mathrm{method}{1}^{\dagger }}}\times 100 $$	

†method1 is current practice (screening all records)

‡method2 is PICo-based title-only screening

## Results

**Fig. 1:**
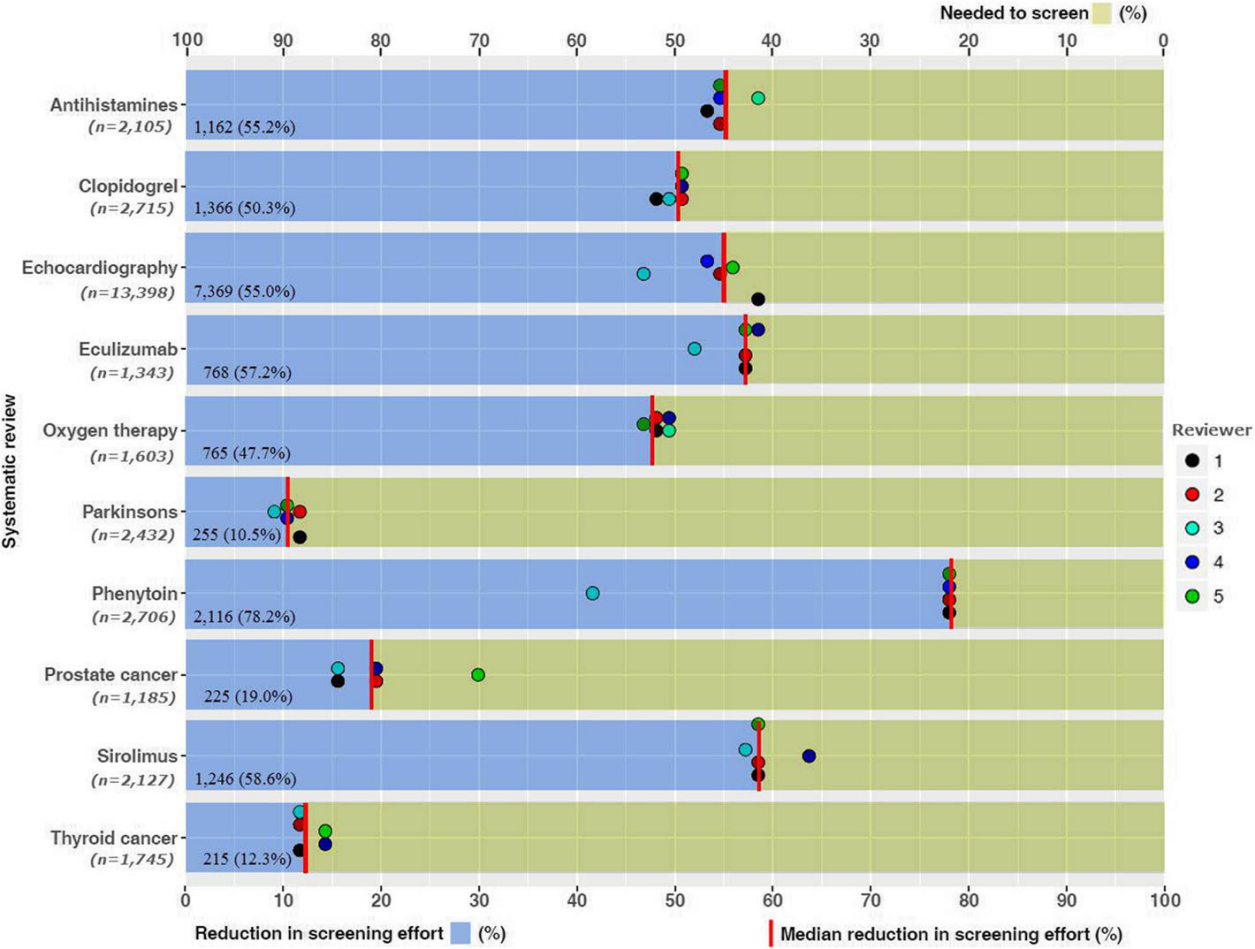
Summary of the median reduction () in screening effort, the individual reviewer reduction in screening effort (coloured dots) and the percentage of citations remaining that are needed to screen across 10 systematic reviews using PICo-based title-only screening

Ten systematic reviews were evaluated with a total of 31,359 records. Reduction in screening effort across the reviews (Fig. 1) ranged from 11% (Parkinson’s review) to 78% (Phenytoin review) with a median reduction in screening effort of 53%. The recall of reports of included studies was 100% in 9 of the 10 reviews (Table 2). In the oxygen therapy review, 4 of 5 reviewers missed the same included study (median recall 67%). Differences in the selection of PICo keywords between reviewers affected the reduction in screening effort, the most notable example being the Phenytoin dataset. Four reviewers obtained similar results with a reduction in screening effort of 78%, whereas one reviewer (reviewer 3) achieved only a 42% reduction in screening effort due to the inclusion of the PICo screening keyword ‘epilepsy’. The focus of treatment was seizure prophylaxis after brain injury.

**Table 2:** Recall for 10 reviews investigated

Review	Recall
Antihistamines	17/17
Clopidogrel	5/5
Echocardiography	51/51
Eculizumab	13/13
Oxygen therapy	2/3*
Parkinson’s	43/43
Phenytoin	6/6
Prostate cancer	29/29
Sirolimus	9/9†
Thyroid cancer	35/35

*One reviewer identified all three studies

†Review included 11 studies, with 2 not retrieved from bibliographic database searches and assumed to be from hand searches and therefore not included in the analysis but would have been retrieved with PICo screening

### Post hoc analysis

**Fig. 2:**
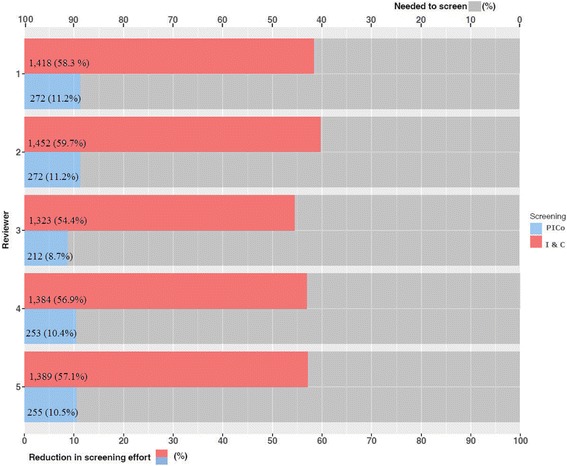
Parkinson’s (*n* = 2432) dataset. Summary of the individual reviewer reduction in screening effort using PICo-based title-only screening () and Intervention- and Comparator-based title-only screening () and the percentage of citations remaining that are needed to screen ()

The minimal reduction in screening effort in the Parkinson’s dataset was principally due to the keyword ‘Parkinson’ retrieving 80% of all records. A post hoc analysis was performed to determine if complete recall could be maintained and reduction in screening effort improved when relying only on keywords for the intervention(s) and comparator(s) but not the participants. Screening without type of participants improved the median reduction in screening effort from 11 to 57%, and the recall of included studies was 100% (Fig. 2).

## Discussion

Our results indicate that PICo-based title-only screening considerably reduces the workload of citation screening, maintains high recall of relevant studies and can be used to expedite scoping searches and rapid reviews.

### Reduction in screening effort

The reduction in screening effort ranged from 11 to 78% with seven of the datasets having a reduction in screening effort above 50%. The two prognostic review datasets (Prostate and Thyroid cancer) had a median reduction in screening effort of 12 and 19%; however, these reviews used a more focused search and are atypical of most search strategies. The post hoc analysis was undertaken because the reduction in screening effort was minimal in the Parkinson’s dataset due to 80% of the citations containing the keyword ‘parkinson’ or variations e.g. ‘parkinsonian’ in the title field, and therefore, the median reduction in screening was only 11%; the post hoc analysis found that restricting the PICo search terms to only the intervention and comparator maintained 100% recall and improved the reduction in screening effort to 57%. This could be a useful strategy to ensure a reduction in screening effort is maintained when a particular PICo term is ubiquitous within a dataset.

The median reduction in screening effort was 53% but varied considerably from 11 to 78%. PICo-based title-only screening may confer minimal time-saving advantages when the reduction in screening is only small (such as 10–20%) as found in the relatively small prostate and thyroid cancer datasets, although knowledge of such scenarios would be difficult to foresee. However, for searches that are not highly focused, even a small reduction in screening effort may be advantageous, especially when datasets are large. In addition, general searches conducted in MEDLINE typically produce over 17,000 citations [6], suggesting that most searches are not highly focused and these would also benefit by applying PICo-based title-only screening. Care must be taken to ensure British and American spellings and suffix variations are incorporated into the keyword screening and that compound terms e.g. ‘transoesophageal echocardiography’ are entered as separate search terms to allow for variations in word order; otherwise, relevant citations could potentially be missed when using PICo-based title-only screening.

### Recall

The recall was 100% in 9 of 10 systematic reviews. One reviewer, a clinician, identified all included studies across the 10 reviews including the oxygen therapy review; however, four reviewers missed the same included study in the oxygen therapy review. ‘Ventilation’ was used in the title as an alternative term for oxygen therapy, and this was not listed in the MeSH database nor found whilst searching other resources, and therefore, subject knowledge was needed to identify the study. Nonetheless, for other datasets, PICo-based title-only screening was reliable.

### Strengths and limitation of the research

The strengths in this study were that 10 datasets were used to test the hypothesis that using PICo-based title-only screening could retrieve all studies that should have been found and reduce the number of citations to screen. Also, the results were reproducible for recall in 9 of 10 datasets, and the methodology is simple and easily implemented by reviewers or information specialists with knowledge of screening and Endnote software. The datasets used were a convenience sample, and reduction in screening effort may differ with different clinical specialities and study designs. Applying PICo screening to datasets with less structured vocabulary, such as reviews of non-drug interventions and in fields such as health services research and the social sciences, is likely to be more challenging. Nonetheless, in this study, the sample of reviews tested included a variety of clinical specialties, different types of interventions and different study designs, such as diagnostic accuracy, prognostic and intervention studies.

### Applicability

The limiting factor for the applicability for screening is the presence or absence of either controlled or consistent vocabulary. The high recall and improvement in the reduction in screening effort was due to the sample datasets using clearly defined terms for (P) clinical conditions, (I) interventions and (C) comparators, but using PICo-based title-only screening where the ontology is less clearly defined (e.g. where there are no MeSH terms indexed) could potentially affect recall; in such scenarios, PICo-based title-only screening may be unsuited for rapid review but would remain useful for scoping searches since identifying all studies is not the objective. This potential for error, however, could be allayed by including topic experts to help compile search terms. However, it has been shown that the retrieval of relevant studies for inclusion can be impaired in rapid reviews when the number of databases searched or the number of screeners is restricted [17]. Similarly, traditional title and abstract screening for systematic review can be imperfect with relevant studies wrongly excluded [18]. This screening methodology could also be applied to systematic review screening where one reviewer examines all records whilst a second reviewer screens the sub-set identified from PICo-based-title screening. Also, the PICo screening methodology could be adapted for *searching* directly in biomedical bibliographic databases.

This study has examined expediting screening on the assumption that titles of articles will include at least one of the PICo components to enable a focused title-based search to identify all relevant studies and minimise the number of citations to screen. Other methods have been developed to expedite screening using semi-automated predictive algorithms that ‘learn’ to distinguish relevant and irrelevant citations [19]. The recall and reduction in screening effort from PICo-based title-only screening are similar to those achieved with semi-automated predictive algorithms [19–21]. However, semi-automated screening algorithms require an initial training set (typically ~ 25% of the total citations) to be manually screened in order to train the algorithm. This step could be expedited by incorporating PICo-based title-only screening to generate a sub-set of citations to automatically train the algorithm and dispense with manual training. Further work is needed to explore how PICo screening can be incorporated into machine learning technologies to further accelerate the training of datasets.

## Conclusion

In the sample of datasets used, PICo-based title-only screening was able to expedite citation screening for scoping searches and rapid reviews by reducing the number of citations to screen but requires a thorough workup of the potential synonyms and alternative terms. Further research which evaluates the feasibility of this technique with heterogeneous datasets in different fields (such as health services research) and intervention types (such as non-drug interventions) would be useful to inform the generalisability of this technique.

## Appendix 1. Example of PICo-based search terms used for screening

Clopidogrel and aspirin versus aspirin alone for stroke prevention: a meta-analysis

Inclusion criteria:

Participants—people with stroke or transient ischaemic attack

Intervention—clopidogrel and aspirin

Comparator—aspirin

**Table:**

PICo	Alternate name	Alternate name	Alternate name
Stroke	Intracranial Embolism and Thrombosis	Intracranial Arteriosclerosis	
Transient ischaemic attack	TIA	Brain Stem Ischemia	Transient Cerebral Ischemia
Clopidogrel	plavix	iscover	
Aspirin	Acetylsalicylic acid	ASA	2-(Acetyloxy)benzoic Acid

## Additional file

Additional file 1: Example of PICo-based search terms used for screening. (DOCX 22 kb)

## Abbreviations

MEDLINE: Medical Literature Analysis and Retrieval System Online
MeSH: Medical subject headings
PICo: Participant, Interventions, Comparators, Outcomes
RSE: Reduction in screening effort
