# Supplementary material for: Expediting citation screening using PICo-based title-only screening for identifying studies in scoping searches and rapid reviews
Source: Syst Rev. 2017 Nov 25;6:233. doi: 10.1186/s13643-017-0629-x (PMC5702220; doi:10.1186/s13643-017-0629-x)
Supplement: Additional file 1: — Example of PICo-based search terms used for screening. (DOCX 22 kb) [file 13643_2017_629_MOESM1_ESM.docx]

**Additional File 1**

**Example of PICo based search terms used for screening:**

**Antihistamines for the common cold**

| **PIC**o **Name** | **Alternate name** | **Alternate name** | **Alternate name** | **Alternate name** |
| --- | --- | --- | --- | --- |
| Common cold/cold | rhinorrhea | coryza | Nasal obstruct/block/congestion | URTI/upper respiratory tract infection |
| antihistamines | Histamine Antagonists | H2-receptor antagonist |  |  |
| brompheniramine | Dimetane | Respa-BR |  |  |
| loratadine | Claritin |  |  |  |
| Cetirizine | Zyrtec |  |  |  |
| fexofenadine | Allegra |  |  |  |
| Triaminic | chlorpheniramine | chlorphenamine |  |  |
| promethazine | Phenergan |  |  |  |
| chlorpheniramine | Tussin | chlorphenamine |  |  |
| Diphenhydramine/ diphenhydramine | Benadryl | nytol |  |  |
| hydroxyzine | Vistaril | Atarax |  |  |
| alimemazine | Vallergan |  |  |  |
| acrivastine | Benadryl |  |  |  |
| clemastine | meclastin | Tavegil |  |  |
| Cyclizine | Marezine |  |  |  |
| cyproheptadine | Periactin |  |  |  |
| chlorphenamine | chlorpheniramine | Tussin |  |  |
| desloratadine | Clarinex |  |  |  |
| Dexchlorpheniramine | Polaramine |  |  |  |
| [Doxylamine](https://amhonline.amh.net.au/chapters/chap-01/antihistamines-01/antihistamines-sedating/doxylamine) | Restavit |  |  |  |
| [Pheniramine](https://amhonline.amh.net.au/chapters/chap-01/antihistamines-01/antihistamines-sedating/pheniramine) | Avil |  |  |  |
| [Trimeprazine](https://amhonline.amh.net.au/chapters/chap-01/antihistamines-01/antihistamines-sedating/trimeprazine) | Nedeltran | Panectyl | Repeltin | Therafene |

**Routine echocardiography in the management of stroke and transient ischemic attack (TIA): a systematic review**

| **PIC**o **Name** | **Alternate name** | **Alternate name** | **Alternate name** | **Alternate name** |
| --- | --- | --- | --- | --- |
| Stroke | apoplexy | Cerebrovascular accident | Brain vascular accident |  |
| Transient ischemic attack | TIA | Vertebrobasilar ischemic attack | Transient Brainstem Ischemia |  |
| Transthoracic echocardiography | TTE | Contrast echocardiography | M-Mode Echocardiography | 2D Echocardiography |
| Transesophageal echocardiography | TEE (TOE) |  |  |  |
| Magnetic resonance imaging | MRI scan | MR tomography | Magnetic resonance imaging |  |
| Transmitral Doppler |  |  |  |  |
| Transcranial Doppler | Transcranial Doppler sonography | |  |  |
| Atrial septal defect | ASD |  |  |  |
| Patent foramen ovale | PFO |  |  |  |
| Atrial shunt | Interatrial shunt |  |  |  |
| septal aneurysm |  |  |  |  |
| atrial septum | Interatrial septum |  |  |  |
| ventricular aneurysm |  |  |  |  |
| left ventricular dysfunction |  |  |  |  |
| ejection fraction |  |  |  |  |
| Cor triatriatum | Tratrial heart | Left atrium subdivided |  |  |
| Mitral valve stenosis | Mitral stenosis |  |  |  |
| Rheumatic mitral valve disease | Rheumatic mitral stenosis |  |  |  |
| Mitral valve regurgitation | Mitral valve insufficiency | Mitral incompetency |  |  |
| Mitral valve prolapse | Floppy mitral valve | Click-murmur syndrome |  |  |
| Aortic valve stenosis | Aortic stenosis |  |  |  |
| Rheumatic aortic valve |  |  |  |  |
| Aortic valve regurgitation | Aortic valve insufficiency | Aortic incompetence |  |  |
| Mitral or aortic valve strands |  |  |  |  |
| ventricular thrombosis / thrombi  atrial thrombosis / thrombi | Ventricular /atrial thrombus | |  |  |
| Left ventricular thrombus / left atrial thrombus | LVT / LAT |  |  |  |
| Apical thrombosis | Apical thrombus |  |  |  |
| Atrial appendage thrombus |  |  |  |  |
| atrial thrombi |  |  |  |  |
| Cardiac tumour/mass | Heart tumor | Cardiac neoplasm |  |  |
| Atrial myxoma |  |  |  |  |
| Papillary fibroelastoma |  |  |  |  |
| Libman-Sacks endocarditis | Verrucous endocarditis | Marantic endocarditis | Nonbacterial thrombotic endocarditis |  |
| Marantic endocarditis |  |  |  |  |
| thrombotic endocarditis |  |  |  |  |
| Valvular vegetation |  |  |  |  |
| Dilated left atrium | Left atrium enlargement | Left atrium dilation |  |  |
| Left atrial enlargement | See above |  |  |  |
| Dilated left ventricle | Left ventricular dilation | Dilated cardiomyopathy |  |  |
| Left ventricle hypertrophy | Left Ventricular Hypertrophy | Left Ventricular enlargement |  |  |
| Left ventricular hypertrophic | See above |  |  |  |
| Aortic aneurysm | Aortic dilation |  |  |  |
| Dilated proximal aorta | Dilation of thoracic aorta | Thoracic aortic aneurysm |  |  |
| Calcification aorta | Aortic valve calcification |  |  |  |
| Aortic dissection |  |  |  |  |
| Left atrial appendage |  |  |  |  |
| Cardiomyopathy | Myocardiopathy |  |  |  |
| Dilated cardiomyopathy |  |  |  |  |
| Left ventricular noncompaction | LVNC |  |  |  |
| Atrial flutter | Auricular flutter |  |  |  |
| Sick sinus syndrome | Sick sinus node syndrome | Sinus node dysfunction | Sinoatrial node disease | SND |

**Eculizumab for atypical haemolytic anaemia (aHUS)**

| **PIC**o **Name** | **Alternate name** | **Alternate name** | **Alternate name** | **Alternate name** |
| --- | --- | --- | --- | --- |
| Atypical haemolytic uremic syndrome | AHUS |  |  |  |
| eculizumab | soliris | alexion |  |  |
| Plasma exchange | PE/PI | Blood transfusion | Exchange transfusion | plasmapheresis |
| Kidney dialysis | Renal dialysis | Hemodialysis (haemodialysis) |  |  |
| Transplantation | transplant | Graft |  |  |

**Oxygen therapy for pneumonia in adults**

| **PIC**o **Name** | **Alternate name** | **Alternate name** | **Alternate name** | **Alternate name** |
| --- | --- | --- | --- | --- |
| Pneumonia | Chest infection | Lower respiratory tract infection | LRTI | CAP |
| Oxygen therapy | Oxygen Inhalation Therapy | ventilation | Continuous positive airway pressure | CPAP |
|  |  |  |  |  |

**A systematic review and network meta-analysis of IPX066 and other common therapies for management of symptoms in patients with advanced Parkinson’s disease**

| **PIC**o **Name** | **Alternate names** | **Alternate names** | **Alternate names** | **Alternate names** |
| --- | --- | --- | --- | --- |
| Parkinson’s disease | Parkinsonism | Paralysis Agitans |  |  |
| IPX066 | Rytary | Carbidopa-Levodopa |  |  |
| Levodopa | Sinemet | Parcopa |  |  |
| benserazide | Benspar | Cenparkin | Levoben | madopar |
| Pramipexole | Mirapex | Medopexol |  |  |
| Ropinirole | Requip | Repreve |  |  |
| Rotigotine | Neupro | Rotigotine |  |  |
| Piribedil | ET 495 | EU 4200 | S 495 |  |
| Rasagiline | Azilect |  |  |  |
| Selegiline | Eldepryl | Zelapar |  |  |
| Zydis | olanzapine | zyprexa |  |  |
| Entacapone | Comtan | Anxopone |  |  |
| Nebicapone | 274925-86-9 | DTXSID30181912 |  |  |

**Phenytoin versus Levetiracetam for Seizure Prophylaxis after brain injury – a meta analysis**

| **PIC**o **Name** | **Alternate name** | **Alternate name** | **Alternate name** | **Alternate name** |
| --- | --- | --- | --- | --- |
| Seizure | Traumatic brain injury | TBI | convulsion |  |
| Phenytoin | dilantin | Sodium phenytek |  |  |
| Levetiracetam | keppra | Spritam |  |  |
|  |  |  |  |  |

**Prevalence of incidental prostate cancer: A systematic review of autopsy studies**

| **PIC**o **Name** | **Alternate name** | **Alternate name** | **Alternate name** | **Alternate name** |
| --- | --- | --- | --- | --- |
| Autopsy | Post-mortem (w/ & w/o the -) | Cadaver |  |  |
| Prostate | Prostatic |  |  |  |
| Cancer | neoplasm | Tumor (tumour) |  |  |
|  |  |  |  |  |

**Use of Sirolimus in Liver Transplant Recipients with Renal Insufficiency: Systematic Review and Meta-Analysis**

| Name **PIC**o | **Alternate name** | **Alternate name** | **Alternate name** | **Alternate name** |
| --- | --- | --- | --- | --- |
| Liver transplant | Hepatic transplant | Liver graft | Hepatic graft |  |
| Sirolimus | rapamycin | Rapamune |  |  |
| tacrolimus | Prograf | Protopic |  |  |
| cyclosporine | Cyclosporin A | CyA NOF |  |  |
| mycophenolate mofetil | CellCept |  |  |  |
| daclizumab | Zinbryta | Zenapax |  |  |
| basiliximab | Simulect | CHI 621 |  |  |
|  |  |  |  |  |

**Prevalence of differentiated thyroid cancer in autopsy studies over six decades: A Meta-analysis**

| **PIC**o **Name** | **Alternate name** | **Alternate name** | **Alternate name** | **Alternate name** |
| --- | --- | --- | --- | --- |
| thyroid | Endocrine gland |  |  |  |
| autopsy | Post-mortem (w/ & w/o the -) | Cadaver |  |  |
| Cancer | neoplasm | Tumor (tumour) | carcinoma |  |
|  |  |  |  |  |
